# A fasting-mimicking environment enhances procaspase-activating compound 1 in 2D and 3D glioma cell models

**DOI:** 10.1080/15384101.2026.2614017

**Published:** 2026-01-16

**Authors:** Kiarn Roughley, Abass Khochaiche, Ari Landstra, Michael Valceski, Carolyn Hollis, Michael Lerch, Stéphanie Corde, Moeava Tehei

**Affiliations:** a Centre for Medical Radiation Physics, University of Wollongong, Wollongong, NSW, Australia; b Molecular Horizons, University of Wollongong, Wollongong, NSW, Australia; c Department of Radiation Oncology, Prince of Wales Hospital, Randwick, NSW, Australia

**Keywords:** Apoptosis, spheroid, PAC-1, glioma, caspase

## Abstract

Glioblastoma multiforme (GBM) is the most common form of malignant brain cancer and is generally approached with palliative intent. Preclinical studies suggest that short-term fasting may be an effective tool for enhancing existing cancer therapies by disrupting the glucose-dependent, oncogenic phenotype of many cancers. In this study, we investigated whether a fasting-mimicking environment (FME) enhances the efficacy of an emerging proapoptotic drug, procaspase-activating compound 1 (PAC-1), in 2D and 3D GBM cell models. Ad libitum food consumption (Fed) and FME conditions were simulated *in vitro* by modifying glucose, ketone and serum concentrations. The FME conditions enhanced PAC-1 in U87-MG, T98G and 9L-GS monolayer experiments by significantly reducing the PAC-1 50% inhibitory concentration (IC_50_), delaying cell growth and increasing apoptosis. Similarly, in the 3D spheroid models, the minimum concentration of PAC-1 required to reduce U87-MG and 9L-GS spheroid area was lower in the FME conditions than the Fed conditions. Additionally, we discovered that serum restriction was primarily responsible for the FME-induced PAC-1 enhancement. These finding are the first to demonstrate that fasting-mimicking conditions sensitize 2D and 3D glioma cell models to PAC-1, supporting the use of short-term fasting as a low-cost and widely accessible strategy for enhancing cancer therapies.

## GRAPHICAL ABSTRACT

**Figure:**
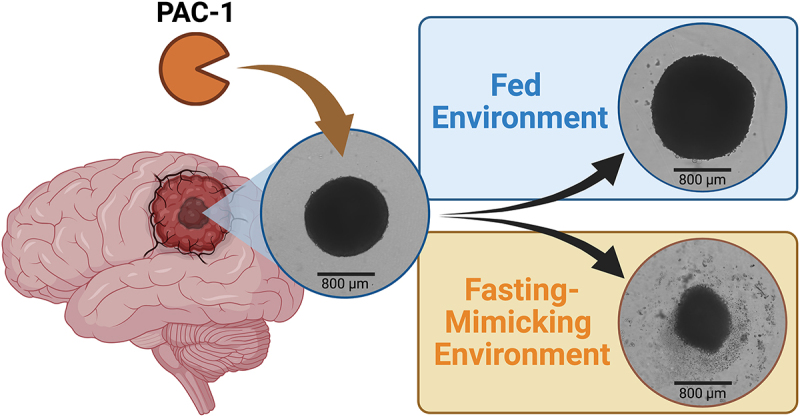

## Introduction

Glioblastoma multiforme (GBM) is the most common form of malignant brain cancer and is generally treated with a combination of surgery, chemotherapy and radiotherapy. Despite these treatment options, GBM remains highly lethal, with a 5-y survival rate of less than 10% and is often approached with palliative intent [1,2]. Therefore, there is an urgent need for novel therapeutic strategies to improve treatment outcomes for GBM patients.

In recent years, the concurrent use of fasting or a fasting-mimicking diet (FMD) alongside cancer treatment has sparked growing interest. Preclinical *in vitro* and *in vivo* studies have shown that short-term fasting (STF) can enhance the effectiveness of certain cancer therapies across multiple cancer types [3–6]. In the context of GBM, Safdie and colleagues have demonstrated that a starvation-mimicking environment sensitizes murine, rat and human glioma cells, but not primary glial cells, to the GBM standard of care chemotherapeutic agent temozolomide (TMZ) [4]. Additionally, the same researchers reported that STF alongside TMZ or radiotherapy treatment further extended survival in a murine glioma model. It has also been shown in humans that STF or FMDs can reduce the severity of common treatment side effects including inflammation, fatigue, weakness and nausea while also improving circadian rhythm regulation [7–11]. This gives rise to the differential stress resistance theory, whereby fasting initiates protective hormetic stress resistance pathways in healthy cells, which are not activated in cancer cells due to their hyperproliferative and oncogenic phenotype [8,9,12]. More broadly, fasting or FMDs offer a relatively safe, low-cost and widely accessible lifestyle intervention that can be combined with cancer modalities and thus warrants investigation [12].

The metabolic phenotype of cancer is often characterized by the upregulation of growth pathways, including aerobic glycolysis (the Warburg effect) and the PI3K-Akt-mTOR (PAM) pathway, alongside resistance to antiproliferative signals. Elevated activity of the PAM axis has been shown to assist cancer cells in evading apoptosis, making it a common site of interest for cancer therapies [13,14]. Fasting offers a nonpharmacological strategy for downregulating the PAM pathway by inducing nutrient and growth factor scarcity, thereby potentially sensitizing cells to apoptosis from certain treatments delivered in parallel [3,15–18]. Specifically, reducing growth factors such as insulin-like growth factor 1 (IGF-1), which is possible through fasting, has been shown to increase caspase-3 activation and the mitochondrial-dependent apoptotic pathway via the release of cytochrome C and the modulation of bcl-2 proteins [19–21]. Additionally, the systemic and pleiotropic nature of fasting targets PAM suppression through multiple inputs, ensuring the downregulation of the pathway and sensitization to apoptosis in cancer cells, despite their hyperproliferative and self-sufficient growth capabilities [3,5,6,12]. However, the extent of the suppression depends on the type, stage, mutation profile and metabolic characteristics of the malignancy. Meanwhile, the ability of healthy cells to initiate stress response pathways during fasting periods appears to result in a relative protective effect against treatment-induced damage [12,22,23]. Therefore, the pro-apoptotic conditions in cancer cells induced by fasting provides a promising strategy for enhancing therapies targeting apoptosis and forms the foundation for this study.

Procaspase-activating compound 1 (PAC-1) is a prime candidate to synergize with fasting as it activates the final stage of apoptosis by chelating zinc and disinhibiting procaspase-3 [24]. As an emerging therapy that has completed phase I clinical trials, PAC-1 is an attractive treatment for GBM because of its ability to cross the blood–brain barrier, high efficacy at relatively low concentrations, synergistic potential with other therapies and because of the high expression of procaspase-3 in many GBM cells [25–27]. Boldingh Debernard and colleagues have previously demonstrated that the addition of epidermal growth factor (EGF) reduces the ability of PAC-1 to activate procaspase-3 and initiate apoptotic cell death in PC-12 cells [20]. This suggests that fasting-induced reductions in growth factors, may provide a favorable physiological state for optimizing PAC-1 efficacy. However, PAC-1 has yet to be evaluated in glioma cells in a fasting-mimicking environment that incorporates physiologically relevant glucose, ketone and serum conditions. Therefore, the aim of this study was to investigate whether a fasting-mimicking environment enhances the efficacy of PAC-1 in 2D and 3D glioma cell models. Given the emerging nature of PAC-1, it is imperative these results be known and considered when designing future clinical trials [25,26].

## Materials and methods

### Cell culture

The human glioblastoma cell lines U87-MG and T98G and the rodent gliosarcoma cell line 9L-GS (ECACC) were maintained in Dulbecco’s Modified Eagle Medium (DMEM) (Gibco, AUS, #11965118) supplemented with 10% fetal bovine serum (FBS) (Cell Sera, AUS) and 1% penicillin–streptomycin (PS) (Gibco, AUS, #15140122). The cells were maintained as a monolayer in T25 cm^2^ or T75 cm^2^ flasks at 37°C in 5% (v/v) CO_2_ and subcultured between 70% and 90% confluence. The doubling times for U87-MG, T98G and 9L-GS during the experiments were (33 ± 3) hours, (31 ± 2) hours and (33 ± 3) hours, respectively. Experiments were conducted within a 4–20 passage range after thawing.

### Fasting mimicking environment (FME)

Short-term fasting was simulated *in vitro* by manipulating glucose, FBS and β-hydroxybutyrate (BHB) (Sigma (via Merck), AUS, #54965) concentrations to create a fasting mimicking environment (FME) medium. Regular DMEM was combined with glucose free DMEM (Gibco, AUS, #11966025) to achieve the desired glucose concentration. The FME medium was characterized by 0.7 g/L glucose, 1% FBS and 3 mM BHB, whereas the ad libitum (Fed) medium consisted of 2.0 g/L glucose, 10% FBS and 0 mM BHB. Both mediums were supplemented with 1% PS. For this study, the FME conditions represent a patient who has undergone water-only fasting for several days, while the Fed conditions reflect a patient who consumes moderate amounts of carbohydrates, fat and protein throughout the day. The concentration of glucose, serum and ketone utilized in the Fed and FME conditions reflect those found in patients whilst in a Fed or STF state, which has also been utilized in previous *in vitro* models of fasting [3–5,28].

### Treatment with procaspase-activating compound 1 (PAC-1)

Procaspase-activating compound 1 (PAC-1) was purchased as a powder (Jomar Life Science, AUS) and prepared using dimethyl sulfoxide (DMSO) and stored at −80°C. The PAC-1 working solutions were created with a DMSO concentration of no greater than 0.2% (for the MTT viability assay) or 0.1% (for the remainder of the experiments), with each experiment consisting of a respective DMSO vehicle control for comparison. The general workflow for all 2D experiments consisted of seeding the cells in the Fed or FME medium for 24 hours prior to the addition of PAC-1. After 24 hours, the media was replaced with relevant media containing PAC-1, and the cells were treated for 72 hours. To ensure that cell growth was maintained throughout the 72 hours of PAC-1 treatment in the FME conditions, the volume of media added during the experiments did not exceed 1 ml/100,000 cells.

### MTT viability assay

The toxicity of PAC-1 in Fed or FME conditions was assessed through the 3-(4,5- dimethylthiazol-2-yl)-2,5-diphenyltetrazolium bromide (MTT) viability assay (Invitrogen, VIC, AUS, #M6494). U87-MG, T98G and 9L-GS cells were treated with PAC-1 in the respective medium in a 96-well plate as described in ‘Treatment with procaspase-activating compound 1 (PAC-1)’. After 72 hours of PAC-1 treatment, the media was replaced with 110 µl of the respective media containing 1 µM MTT and incubated for 2–4 hours at 37°C and 5% (v/v) CO_2_ to form the tetrazolium salts. Following incubation with MTT, the 96-well plate was centrifuged at 380 ×g for 5 minutes and the well volume was replaced with 200 µl of dimethyl sulfoxide (DMSO) (Sigma Aldrich (via Merck), AUS) to solubilize the tetrazolium salts. The plate was left in the dark at room temperature for 10 minutes and then shaken for 5–10 minutes prior to reading the absorbance of each well at 540 nm using the FlexStation 3 microplate reader system (Molecular Devices, USA). The half-maximal inhibitory concentration (IC_50_) was determined with GraphPad Prism V10 (GraphPad Software, Boston, MA, USA) using a normalized dose–response curve fit with variable slope.

### Cell growth during PAC-1 treatment and recovery

The growth of the cells during PAC-1 treatment and during recovery was measured in a 96-well microplate (Corning® via Sigma (via Merck), AUS, #CLS3599) using the Sartorius IncuCyte S3 (Sartorius AG, Göttingen, Germany) at 10× resolution. For each well, 9 images were taken in a 3 × 3 grid pattern every 6 hours and used to determine the confluence of each well using the Sartorius IncuCyte S3 software. The cells were grown and treated in the respective conditions as described in ‘Treatment with procaspase-activating compound 1 (PAC-1)’. Following 72 hours of PAC-1 treatment, the cells were subcultured into a new 96-well microplate in Fed media and maintained for 10 d to assess recovery, with the media replaced every 3 d.

#### AnnexinV-FITC and propidium iodide (PI) apoptosis assay

The cells were grown and treated in the appropriate medium, as described in ‘Treatment with procaspase-activating compound 1 (PAC-1)’, in 12-well plates (Corning® via Sigma (via Merck), AUS, #CLS3513) and processed according to the manufacturer’s instructions. Briefly, following 72 hours of treatment, the cells were harvested using trypsin-EDTA, and 200,000 cells were centrifuged at 380 ×g for 5 minutes at room temperature. After centrifugation, the samples were resuspended in ice-cold phosphate-buffered saline (PBS) (Ca^2+^ and Mg^2+^ free) (Gibco, AUS, #14190144) and then centrifuged at 380 ×g for 5 minutes at room temperature. A total of 100,000 cells were then incubated in 100 µl of Annexin-V binding buffer solution [10 mM HEPES, 140 mM NaCl, 2.5 mM CaCl2; pH = 7.4] (Invitrogen, VIC, AUS) with 5 µl of Alexa-488-conjugated Annexin-V (Invitrogen, VIC, AUS) and 5 µl of Propidium Iodide (PI) (1 mg/ml) (Sigma via Merck, AUS) at room temperature for 15–20 minutes. The cells were analyzed within 1 hour on the BD LSR Fortessa^TM^ X-20 flow cytometer (BD Biosciences, Franklin Lakes, NJ, USA) using the 488 nm excitation laser (detection range 525 ± 25 nm) and 610 nm excitation laser (detection range 610 ± 10 nm) to measure Annexin-V and PI, respectively. Each run consisted of a minimum of 10,000 events. Finally, the percentage of live, early apoptotic, late apoptotic and necrotic cell populations relative to the total number of events per sample were identified using a quadrant gate in FlowJo software (Tree Star, USA).

### 3D spheroid growth

U87-MG and 9L-GS spheroids were created, treated with PAC-1 and recovered in 96-well Corning Ultra-Low Attachment (ULA) plates (Corning® via Sigma (via Merck), AUS, #CLS3471). The FME medium failed to support the growth of T98G in a 3D spheroid model and thus was not possible to include as a 3D model in this study. To create the spheroids, 4000 cells were seeded in a 96-well ULA plate, centrifuged at 300 ×g for 5 minutes at room temperature, and then allowed to grow undisturbed for 4 d in Fed media. The working volume for each well was 300 µl. After 4 d, the Fed media was replaced with either Fed or twice with a gradient media consisting of 0.5 g/L glucose, 0% FBS and 3.4 mM BHB in order to create the desired FME conditions from the Fed medium without disturbing the spheroids. A single media change to convert from the Fed to FME medium was not possible due to the working volume of each well. The double media change was separated by 1 hour and 5 minutes of rocking after and prior to the initial and second media change to ensure adequate mixing. Following 24 hours, the media was refreshed with Fed or FME media containing PAC-1. After 3 d of PAC-1 treatment, each well was replaced twice with Fed media to ensure that the PAC-1 concentration was less than 1 µM – the minimum threshold determined to illicit a response in both cell lines based on preliminary results. Each media change was separated by 1 hour and rocking as described previously. The Fed media was then replaced every 3 d, up to 6 d post treatment or 13 d after the initial seeding. To monitor the spheroid response, images were taken at 12-hour intervals in the Sartorius Incucyte S3 at 4x magnification. The spheroid area was extracted from each image via ImageJ [29] using binary processing, water shedding and a size threshold of 0.1 mm during image analysis.

### Statistical analysis

The results are expressed as the mean ± SEM with a minimum of three independent repeats. All Statistical analyses were performed in GraphPad V10 using an unpaired t-test with Welch’s correction. Confirmation that the data follows a normal distribution was confirmed via the Shapiro–Wilk test prior to applying the Welch’s t-test. Significance levels are presented as *p* < 0.05 (*), *p* < 0.01 (**) or *p* < 0.001 (***).

## Results

### A fasting-mimicking environment (FME) sensitizes U87-MG, T98G and 9L-GS cells to PAC-1 by reducing cell viability

**Figure 1.**
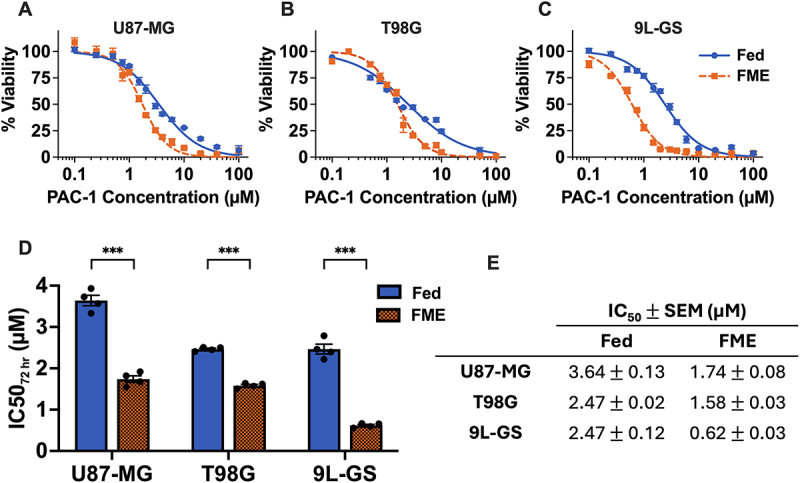
Cell viability of U87-MG, T98G and 9L-GS after 72 hours of PAC-1 treatment in Fed or FME conditions. (A-C) Dose–response curves of U87-MG, T98G and 9L-GS cells following 72 hours of incubation with PAC-1 in the Fed or FME medium. (D-E) The PAC-1 concentration required to reduce cell viability to 50% (IC_50_ value) was derived using a normalized dose–response curve fit with variable slope in GraphPad prism V10. All data is presented as the mean ± SEM of four independent repeats with a minimum of four technical replicates. Statistical significance between the Fed and FME IC_50_ values was determined via unpaired t-test with Welch’s correction; *p* < 0.05 (*), *p* < 0.01 (**) or *p* < 0.001 (***).

To initially screen whether the Fed or FME conditions influenced PAC-1 treatment, we measured the viability of U87-MG, T98G and 9L-GS cells after 72 hours of PAC-1 treatment in the respective media. In all three cell lines, the FME conditions increased PAC-1 toxicity, as demonstrated by a leftward shift in the dose–response curves relative to the population treated in Fed conditions (Figure 1(A-C)). To quantify the PAC-1 enhancement, the concentration of PAC-1 required to reduce cell viability to 50% (IC_50_) was determined on GraphPad V10 using the dose–response curves and compared in Figure 1(D-E). Across all three cell lines, the IC_50_ value for the FME population was significantly lower than that of the Fed population.

The largest PAC-1 enhancement was recorded in 9L-GS with the FME conditions reducing the PAC-1 IC_50_ by 75% compared to the Fed IC_50_ value (*p* = 0.0004). The FME conditions also decreased the PAC-1 IC_50_ in U87-MG and T98G by 52% (*p* < 0.0001) and 36% (*p* < 0.0001), respectively, despite the overlap in the shoulders of the T98G dose–response curves (Figure 1(C)). These data suggest that the FME conditions sensitize glioma to PAC-1 during 72 hours of treatment.

### The FME medium enhances the anti-proliferative and pro-apoptotic effects of PAC-1

**Figure 2.**
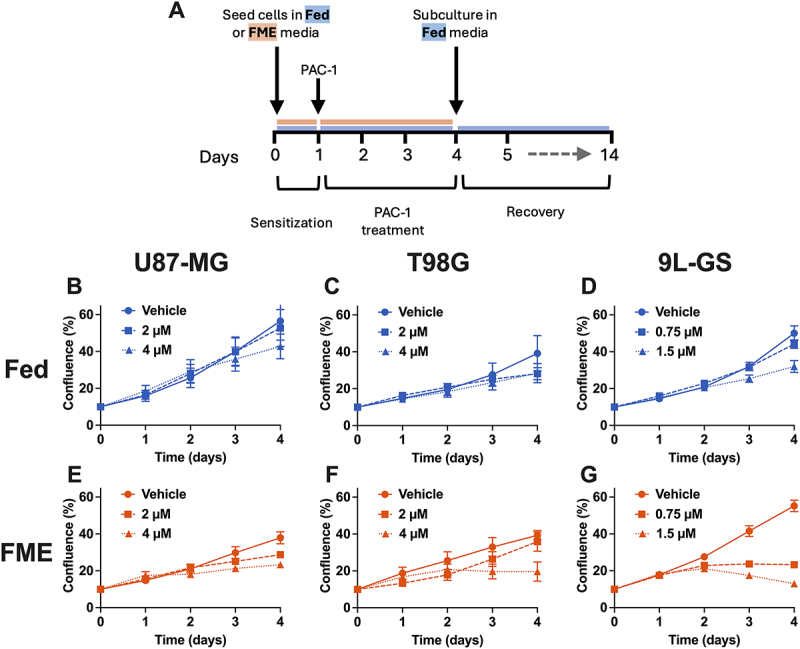
Growth of U87-MG, T98G and 9L-GS during PAC-1 treatment in Fed or FME conditions. (A) The general workflow for all 2D cell experiments, with PAC-1 treatment occurring between days 1 to 4. Cell growth was measured during PAC-1 treatment in Fed (B-D) or FME media (E-G) using the Sartorius IncuCyte S3 live cell imager at 10 × magnification. The concentration of PAC-1 for treatment corresponds to the IC_40_ and IC_20_ of the FME population, determined using Figure 1. The growth during recovery from PAC-1 is shown in Figure S1. The results are presented as the mean ± SEM of at least three independent experiments containing a minimum of four technical replicates.

To gain a more comprehensive understanding of how the FME conditions influence the cellular response to PAC-1, we measured confluence throughout the PAC-1 treatment regimen (Figure 2) and during recovery in Fed conditions (Fig. S1) as an indicator of cell growth. A diagram of the general workflow for all 2D experiments is depicted in Figure 2(A). The concentrations of PAC-1 tested corresponds to the 40% and 20% inhibitory concentration for the FME population specifically of each cell line, as determined in GraphPad using Figure 1. The corresponding PAC-1 40% and 20% inhibitory concentration for the FME population will be referred to as the IC_40_ and IC_20_, respectively, throughout this study. The FME IC_40_ was chosen as it was the lowest PAC-1 concentration that could be explored before completely arresting cell confluence in the 9L-GS FME population (Figure 2(G)). Additionally, the inclusion of the FME IC_20_ allowed us to investigate how a higher PAC-1 concentration further influenced treatment response. The IC_40_ and IC_20_ was calculated as 2 µM and 4 µM, respectively, for both U87-MG and T98G, indicating similar treatment response across both cell lines in the FME conditions. Meanwhile for 9L-GS, the corresponding PAC-1 IC_40_ and IC_20_ was determined as 0.75 µM and 1.5 µM, respectively.

PAC-1 began to produce noticeable effects on confluence 24 hours into treatment and continued for up to 72 hours of treatment (Figure 2(B-G)). We found that the FME IC_20_ concentration was sufficient to completely arrest confluence in U87-MG and T98G, while reducing confluence in 9L-GS (Figure 2(E-G)). In contrast, treating the Fed populations with the same IC_20_ concentrations failed to arrest confluence as confluence continued to grow during treatment, although at a delayed rate to the vehicle control (Figure 2(B-D)). The increase in confluence of U87-MG and T98G during recovery from PAC-1 in Fed conditions showed no large variations between the populations treated in Fed or FME conditions with the same PAC-1 concentration (Fig. S1A, B, D, E). Alternatively, the 9L-GS treated in Fed conditions followed the same growth curve (Fig. S1C), irrespective of treatment concentration, whereas the 9L-GS cells treated in the FME conditions showed a dose-dependent delay in recovery (Fig. S1F). These findings indicate that the FME-induced PAC-1 enhancement does not influence recovery in U87-MG and T98G but does influence recovery in 9L-GS.

Since the primary objective of PAC-1 treatment is to induce apoptotic cell death, we sought to investigate if the arrest in confluence when treating with PAC-1 in the FME conditions was primarily driven by increasing apoptosis. Apoptotic cell death was measured 72 hours after treatment with the PAC-1 IC_40_, as the IC_40_ was the lowest concentration that could be tested before arresting confluence in the FME population of 9L-GS and interfering with the experimental procedure (Figure 2(G)).

**Figure 3.**
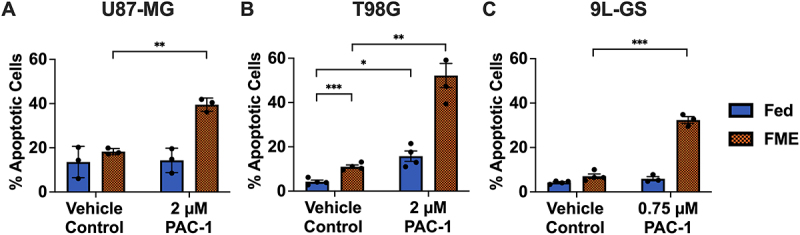
Apoptosis in U87-MG, T98G and 9L-GS following 72 hours of PAC-1 treatment in Fed or FME conditions. Percentage of apoptotic cell death in U87-MG (A), T98G (B) and 9L-GS (C) in response to treatment with a PAC-1 corresponding to the FME IC_40_, as determined in Figure 1. The breakdown of necrotic, early and late apoptotic populations are shown in Figure S2. Apoptosis was measured using Annexin-V and propidium iodide staining with flow cytometry with a minimum of 10,000 events per run. The results are presented as the mean ± SEM of at least three independent repeats. The results were analyzed using an unpaired t-test with Welch’s correction; *p* < 0.05 (*), *p* < 0.01 (**) or *p* < 0.001 (***).

The results in Figure 3 show that the FME conditions significantly increased the ability of PAC-1 to induce apoptosis above the FME baseline in U87-MG, T98G and 9LG-GS by 40%, 52% and 42%, respectively. Meanwhile, treatment with PAC-1 in Fed conditions failed to increase apoptosis in U87-MG and 9L-GS. Although there was a statistically significant 12% increase in apoptosis when treating T98G cells with PAC-1 in Fed conditions (*p* = 0.0007), this increase was still lower than the 52% increase demonstrated in the treated FME population (*p* = 0.0033). The increase in apoptosis in the treated Fed population is likely due to the smaller window of enhancement for T98G compared to U87-MG and 9L-GS, as demonstrated in Figure 1. Overall, the apoptotic results shown in Figure 3 demonstrates that the FME medium lowers the minimum threshold of PAC-1 concentration required to activate apoptosis, which is a desirable result for the clinical setting. Moreover, these data also suggests that the stalling of confluence observed from PAC-1 treatment in FME conditions (Figure 2) is likely attributed to apoptotic cell death, rather than solely a reduction in cell proliferation.

Further analysis revealed that, across all three cell lines, the FME medium primarily increased early apoptotic events rather than increasing late apoptosis or necrosis (Fig. S2). This indicates that PAC-1 continues to activate the apoptotic pathway up to 72-hours after the commencement of treatment. This is in agreement with the growth curve results (Figure 2), showing that PAC-1 continuously impacts confluence between 24 and 72 hours of treatment.

### PAC-1 induces greater reductions in 3D spheroid area in FME conditions

After confirming the FME-induced PAC-1 enhancement in three 2D glioma cell models through the viability, growth and apoptosis assays, we investigated whether this effect translated to a 3D spheroid model using the human U87-MG and rodent 9L-GS cell lines. The FME conditions failed to support the growth of T98G in a 3D spheroid model and thus was not possible to include as a 3D cell model using the methods in this study. However, investigating two spheroid models is sufficient for validating the PAC-1 enhancement in a more clinically relevant 3D tumor model.

**Figure 4.**
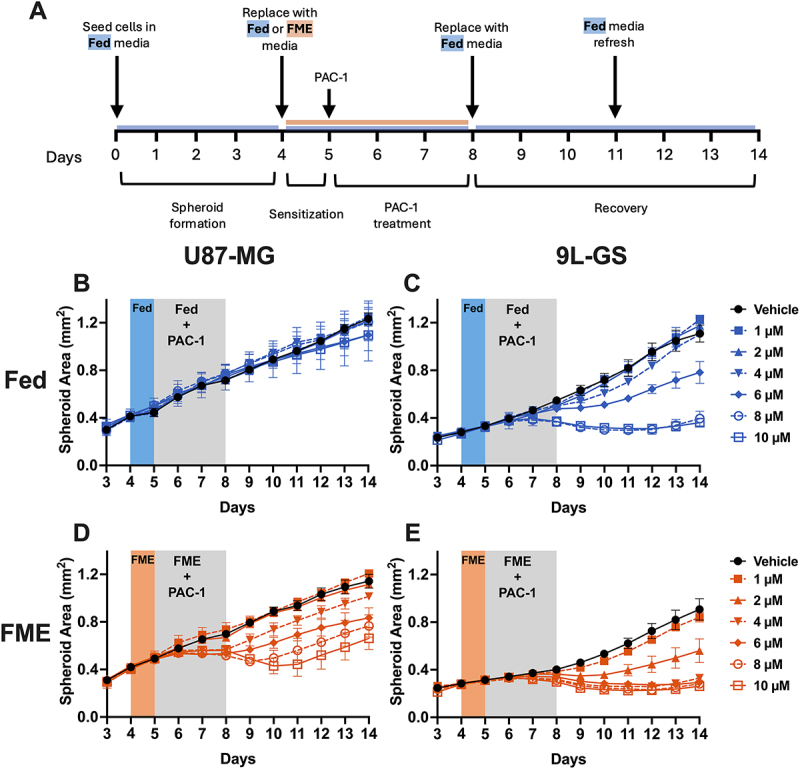
Growth of U87-MG and 9L-GS spheroids during PAC-1 treatment and recovery in Fed conditions. (A) General workflow for the spheroid experiment including relevant media conditions. All spheroids were grown from 4000 cells in Fed conditions, pre-exposed and treated with PAC-1 for 72 hours in Fed (B, C) or FME media (D, E) and then maintained in Fed conditions during recovery. Representative images taken 72 hours after treatment (day 8) and 72 hours into recovery (day 11) are shown in Figure S3 and Figure S4 for U87-MG and 9L-GS, respectively. The spheroid area was calculated on ImageJ using images taken with the Sartorius IncuCyte S3 live cell imager at 4× magnification. The results are displayed the mean ± SEM of at least three independent repeats with each experiment containing a minimum of four technical replicates.

A similar treatment protocol used in the monolayer experiments was adopted for the spheroid experiments, with the addition of a preliminary 4-d seeding in Fed conditions to allow the formation of spheroids (Figure 4(A)). Allowing the spheroids to form in Fed conditions provided a consistent baseline across Fed and FME populations which better represents patients diagnosed with GBM who do not already follow an established fasting protocol. The general workflow proceeded after 4 d post seeding and consisted of 24 hours of pretreatment exposure to the Fed or FME conditions, followed by 72 hours of PAC-1 treatment in the respective medium, and then recovery in Fed conditions. As shown in Figure 4(B-E), the minimum concentration of PAC-1 required to reduce the spheroid area relative to the corresponding vehicle control was lower in the spheroids treated in the FME conditions. Specifically, in U87-MG, 4 µM PAC-1 was sufficient to reduce the spheroid area during treatment in FME conditions, while 10 µM failed to notably influence the area of the spheroids treated in Fed conditions (Figure 4(B, D)). Similarly, the minimum tested concentration required to reduce the 9L-GS spheroid area during PAC-1 treatment was 2 µM and 4 µM for the FME and Fed populations, respectively (Figure 4(C, E)). These findings support the 2D model results, confirming the ability of the FME conditions to enhance PAC-1 in U87-MG and 9L-GS 3D glioma models. Importantly, the validation of the PAC-1 enhancement in a 3D model that better reflects the architecture of clinical tumors improves the clinical applicability of the results presented in this study.

### Serum deprivation is responsible for the PAC-1 enhancement in the FME conditions

**Figure 5.**
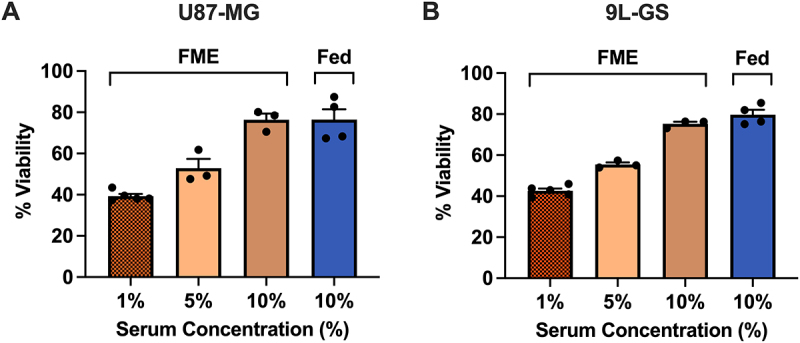
Serum replenishment attenuates the FME-induced PAC-1 enhancement by restoring U87-MG and 9L-GS cell viability. Cell viability was measured in U87-MG (A) and 9L-GS (B) after 72 hours of PAC-1 treatment in FME conditions (0.7 g/L glucose, 3 mM BHB) containing 1, 5 or 10% serum to match the serum composition of the Fed conditions (2.0 g/L glucose, 0 mM BHB, 10% serum). The concentration of PAC-1 tested corresponds to the IC_40_ of the 1% serum FME population, determined using Figure 1. The results are normalized to the vehicle control grown the same respective media and presented as the mean ± SEM of at least three independent experiments containing a minimum of four technical replicates.

The supplementation of epidermal growth factor (EGF) has previously been shown to reduce the ability of PAC-1 to activate procaspase-3 and induce apoptosis [20]. This suggests that PAC-1 could potentially be enhanced by limiting serum which contains growth factors and thus explain the results shown in Figures 1–4. To test the hypothesis that restricting serum was responsible for the PAC-1 enhancement, cell viability of U87-MG and 9L-GS was measured 72 hours after PAC-1 treatment with the FME IC_40_ for each cell line in varying serum concentrations. The results in Figure 5(A,B) demonstrate a dose-dependent restoration of cell viability from PAC-1 as serum was restored from 1% to 10% in the FME medium. These data suggest that restricting serum is responsible for enhancing PAC-1. Interestingly, the differences in glucose and ketone concentrations across the Fed (2.0 g/L glucose, 0 mM BHB) and FME (0.7 g/L glucose, 3 mM BHB) conditions appeared to have no significant influence on cell viability once the serum concentration was restored to 10% in the FME media. These findings indicate that dietary interventions focused on suppressing growth factors may provide a useful approach for improving the therapeutic efficacy of PAC-1 in the clinical setting.

## Discussion

GBM remains an incurable disease, with survival rates showing only marginal improvements in recent decades [1,2]. Therefore, innovative therapeutic approaches are urgently needed in order to meaningfully increase patient long-term survival. The aim of this study was to investigate whether fasting mimicking conditions could offer a non-pharmacological strategy for enhancing the efficacy of PAC-1 in glioma. This study demonstrates that FME conditions enhance PAC-1 in U87-MG, T98G and 9L-GS 2D glioma cell models by reducing cell viability, delaying cell growth and increasing apoptotic events. This enhancement was also observed in U87-MG and 9L-GS 3D spheroid models, where the FME medium induced greater PAC-1-mediated reductions in the spheroid area compared to the spheroids treated in the Fed conditions. Additionally, we revealed that the FME-induced PAC-1 enhancement was mediated through serum reduction and independent of the glucose or β-hydroxybutyrate ketone concentrations used in both culture mediums. Moreover, sensitivity to PAC-1 and the magnitude of the PAC-1 enhancement mediated by the FME conditions varied between the cell lines when comparing the IC_50_ values. In agreement with previous findings in 2D models, 9L-GS and then T98G cells were the most responsive to PAC-1 in Fed conditions, followed by U87-MG [30]. The largest FME-mediated enhancement according to the IC_50_ results was seen in 9L-GS, followed by U87-MG and then T98G. The consistency of the results between the rodent and human glioma cell lines implies that improving PAC-1 efficacy through the FME conditions is conserved across species and not GBM cell line specific. Since this study investigates whether the FME conditions enhance PAC-1 in glioma only, future research involving a normal glial cell line is required to determine if the PAC-1 enhancement also occurs in normal cells or if they are protected by the differential stress response theory [4].

The ability of serum replenishment to restore 2D cell viability in treated U87-MG and 9L-GS in a dose-dependent manner suggests that serum starvation drives the PAC-1 enhancement in this study. This effect likely arises from the depletion of growth factors present in the serum, such as EGF, which has been shown to attenuate PAC-1-induced apoptosis in PC12 cells [20]. Additional growth factors in serum include platelet-derived growth factor (PDGF) and insulin-like growth factor 1 (IGF-1), the latter of which has become a popular topic for investigation in cancer [31]. Serum or growth factor withdrawal also promotes the mitochondrial-dependent apoptotic pathway via suppression of the PAM axis, resulting in the upregulation of certain proapoptotic proteins, such as p53-upregulated modulator of apoptosis (PUMA) [19,32]. Similarly, PAC-1 has also been shown to increase PUMA and suppress the activation of certain growth factor receptors, including vascular epidermal growth factor (VEGF), fibroblast growth factor (FGF) and EGF, resulting in the suppression of downstream kinases present in the PAM pathway [33,34]. The ability of PAC-1 and the FME conditions to collectively suppress the PAM growth pathway and promote mitochondrial death signaling may explain one of the mechanisms through which the FME conditions enhance PAC-1. The starvation conditions are also likely to upregulate autophagy via nutrient deprivation, potentially contributing to the altered treatment response [35]. These pathways should be explored in future mechanistic studies investigating the potential synergy between STF and PAC-1. Furthermore, the influence of serum on PAC-1 efficacy highlights the need to ensure consistent experimental serum conditions for future *in vitro* studies involving PAC-1 to maintain the accuracy and reliability of the results.

Considering that the enhancement of PAC-1 was independent of the glucose and ketone concentrations, future clinical dietary protocols aimed at optimizing PAC-1 treatment may choose to focus primarily on reducing growth factors at the site of the malignancy. While short-term fasting (STF) is one of the most successful lifestyle strategies for reducing certain circulating growth factors, caloric restriction (CR) may offer a less potent yet more sustainable alternative, as CR exceeding 50% caloric baseline needs is capable of reducing IGF-1 levels [36]. Moreover, the fasting-mimicking diet (FMD) developed by Longo and colleagues, offers another approach to simulate fasting through a low-calorie, low-carbohydrate and low-protein meal plan [7]. Personalized dietary interventions, such as these, are relatively safe, cost-effective and empower the patients to take an active role in their care and treatment. Additionally, dietary approaches including STF and FMD would ideally be paired with refeeding periods that incorporate highly nutritious foods to enhance overall diet quality and support the patient well-being. Importantly, the specific methods and strategies that will be applied for each patient would be determined on a case-by-case basis in order to maximize compliance and minimize potential side effects, such as unintended weight loss.

Although this study provides promising findings that warrant further investigation, the reliance on *in vitro* models with established cell lines inevitably limits the clinical applicability of the results. Most notably, the dynamic and pleiotropic effects of feeding and fasting cannot yet accurately be captured with *in vitro* cell models. Instead, the current practice is to manipulate primarily glucose, serum and to a lesser extent ketone concentrations to mirror those found in patients undergoing STF or maintaining regular food intake – as was done for this study [28]. Additionally, while 3D spheroids offer improved translational relevance compared to 2D models, their lack of cell heterogeneity throughout the spheroid and relatively small size fail to replicable clinically relevant characteristics of GBM tumors. However, the significant variability in size, morphology and composition across clinical tumors presents a challenge in developing a broadly applicable spheroid model. Collectively, the limitations of this study may obscure clinically relevant insights, such as exploring the fasting-induced differential stress resistance (DSR) between healthy and malignant cells or how the immune system under fasting conditions may influence the treatment response [12,22,23]. Nevertheless, the methods and results presented in this study provide an essential foundation for guiding future research exploring these influences in subsequent *in vitro*, animal and human studies.

The addition of therapies known to synergize with PAC-1 or with STF alone, such as temozolomide (TMZ) or radiotherapy, could provide a supra-additive treatment response when combined in a multimodal treatment regimen [4,30]. Additionally, clinically available therapies targeting growth factor receptors and the PAM axis, such as Osimertinib, Bevacizumab (Avastin) and metformin, may offer a pharmacological approach for enhancing PAC-1 through similar methods to STF. This potential for PAC-1 to synergize with STF and established therapies presents a promising avenue for developing an optimal multimodal treatment strategy for GBM and other malignancies. Overall, the results presented in this study are the first to demonstrate that fasting-mimicking conditions enhance the efficacy of PAC-1 in 2D and 3D glioma cell models, suggesting that STF could serve as a personalized, noninvasive, widely accessible and cost-effective strategy for enhancing PAC-1 therapy.

## Supplementary Material

Fig_S4.pdf

Fig_S3.pdf

Supplementary_Figures_KR.docx
