# Supplementary figures and images for: A fasting-mimicking environment enhances procaspase-activating compound 1 in 2D and 3D glioma cell models
Source: Cell Cycle. 2026 Jan 16;25(1):1–12. doi: 10.1080/15384101.2026.2614017 (PMC12915821; doi:10.1080/15384101.2026.2614017)

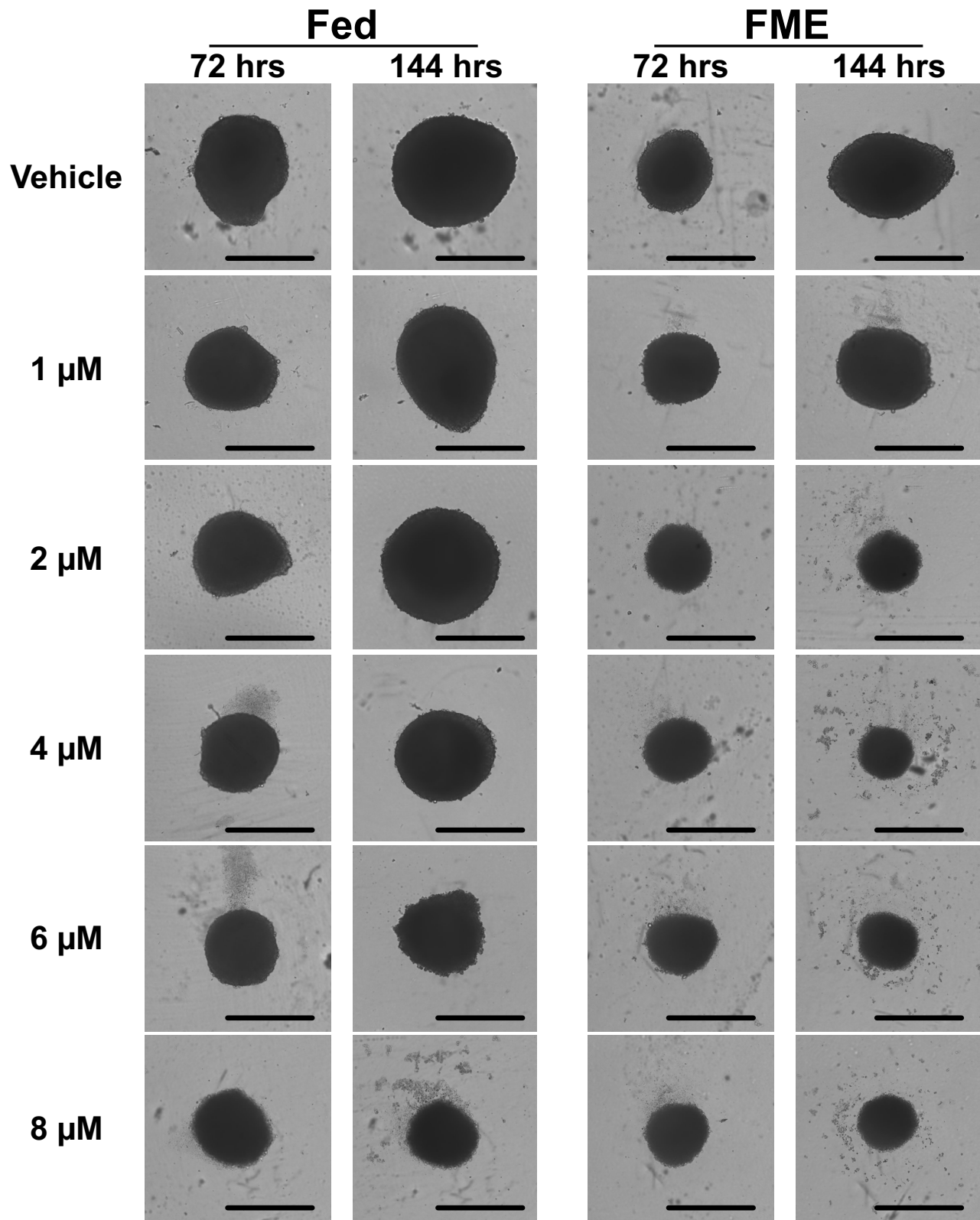

Supplement: Fig_S4.pdf [file KCCY_A_2614017_SM2649.pdf]

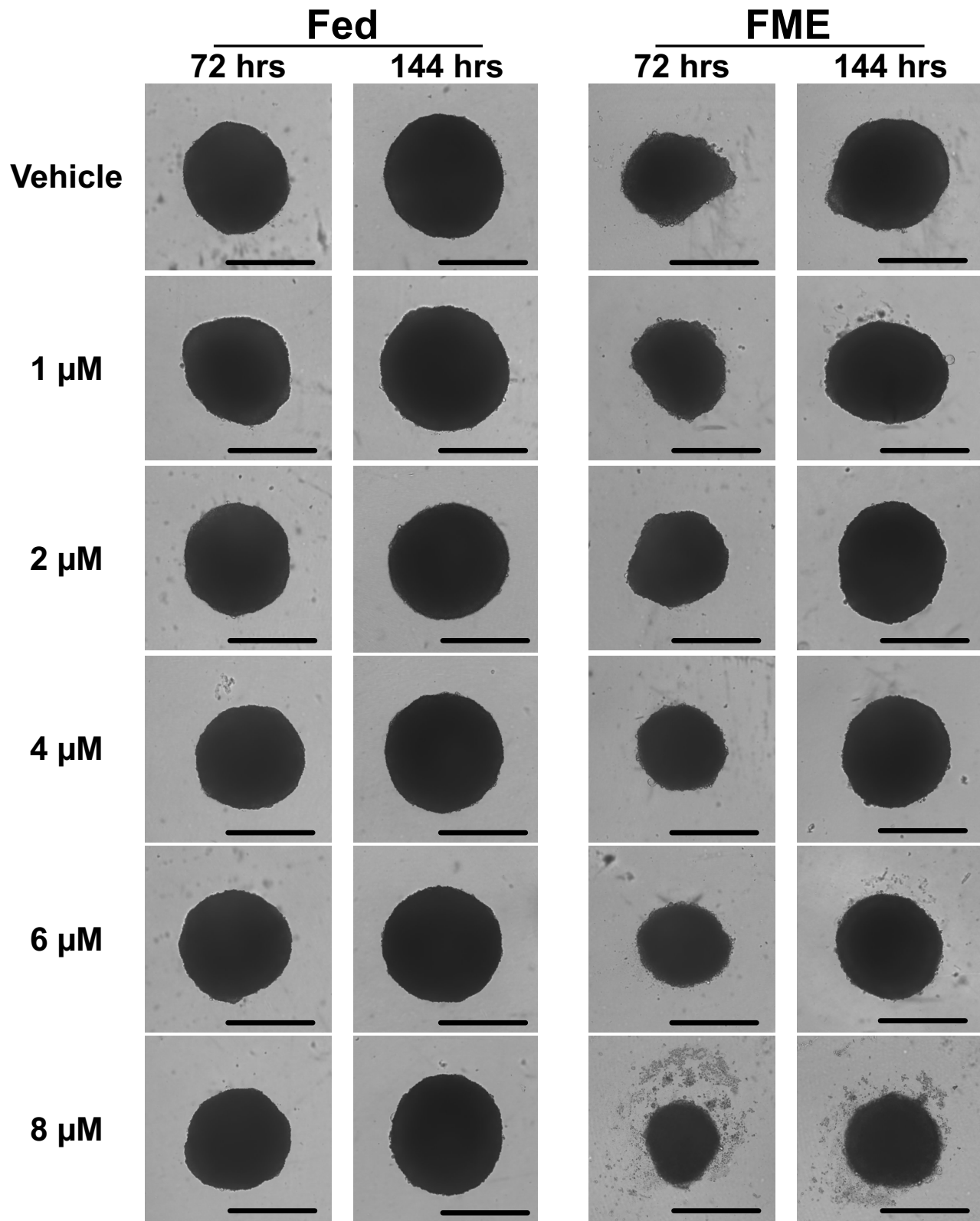

Supplement: Fig_S3.pdf [file KCCY_A_2614017_SM2648.pdf]
