# Supplementary material for: A fasting-mimicking environment enhances procaspase-activating compound 1 in 2D and 3D glioma cell models
Source: Cell Cycle. 2026 Jan 16;25(1):1–12. doi: 10.1080/15384101.2026.2614017 (PMC12915821; doi:10.1080/15384101.2026.2614017)
Supplement: Supplementary_Figures_KR.docx [file KCCY_A_2614017_SM2647.docx]

**Supplementary Figures**

**
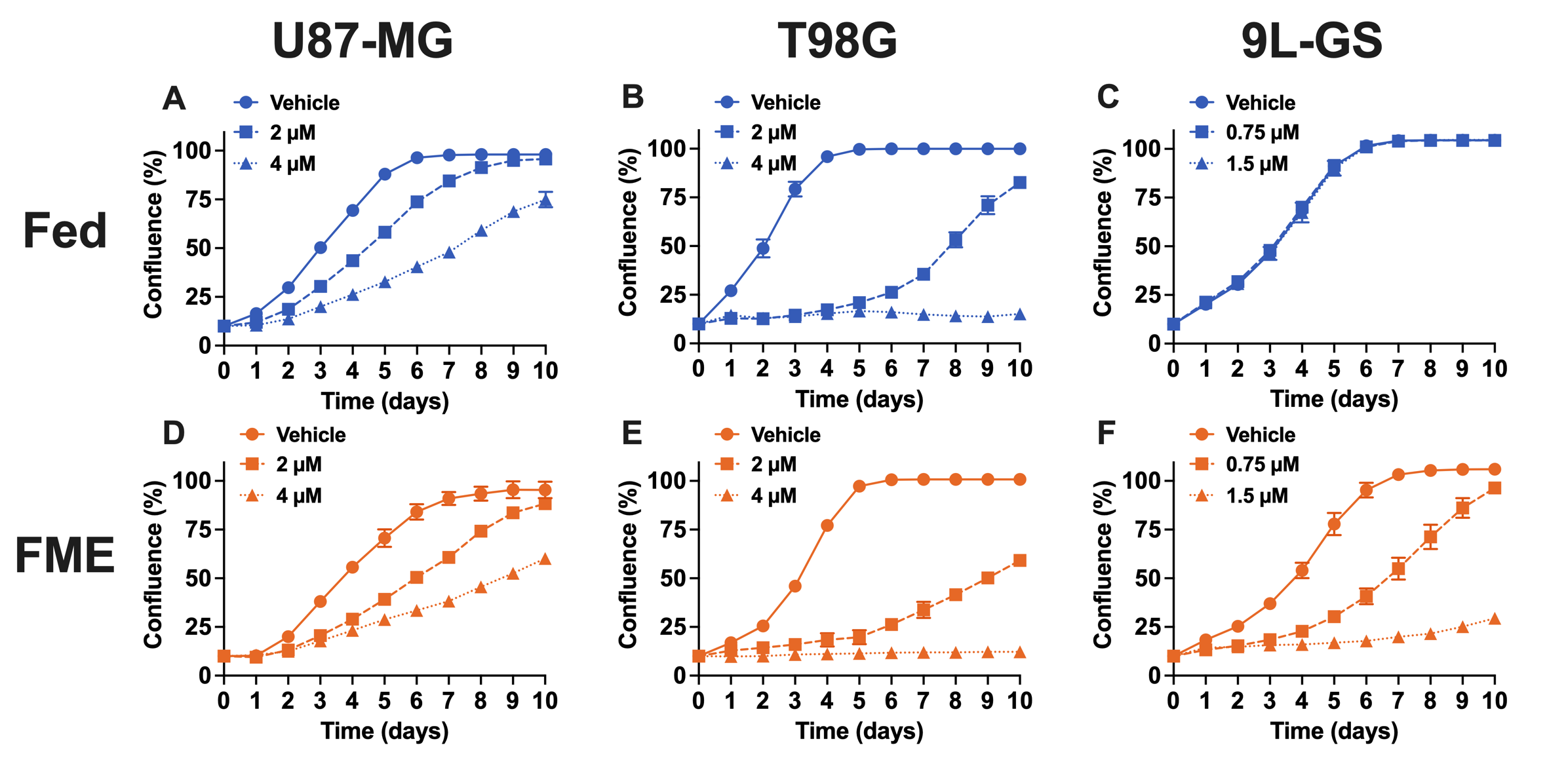
**

**Fig. S1. Growth of U87-MG, T98G and 9L-GS during recovery in Fed conditions from PAC-1 treatment in Fed or FME conditions.** The cells were treated in Fed (**A-C**) or FME (**D-F**) conditions for 72 hours with the concentration corresponding to the FME IC_40_ and IC_20_ determined using Fig. 1. Following 72 hours of treatment, the cells were subcultured in Fed media. Cell confluence was determined using the Sartorius IncuCyte S3 live cell imager at 10 x magnification with the results presented as the mean $\pm$ SEM of at least three independent repeats containing a minimum of four technical replicates.

**
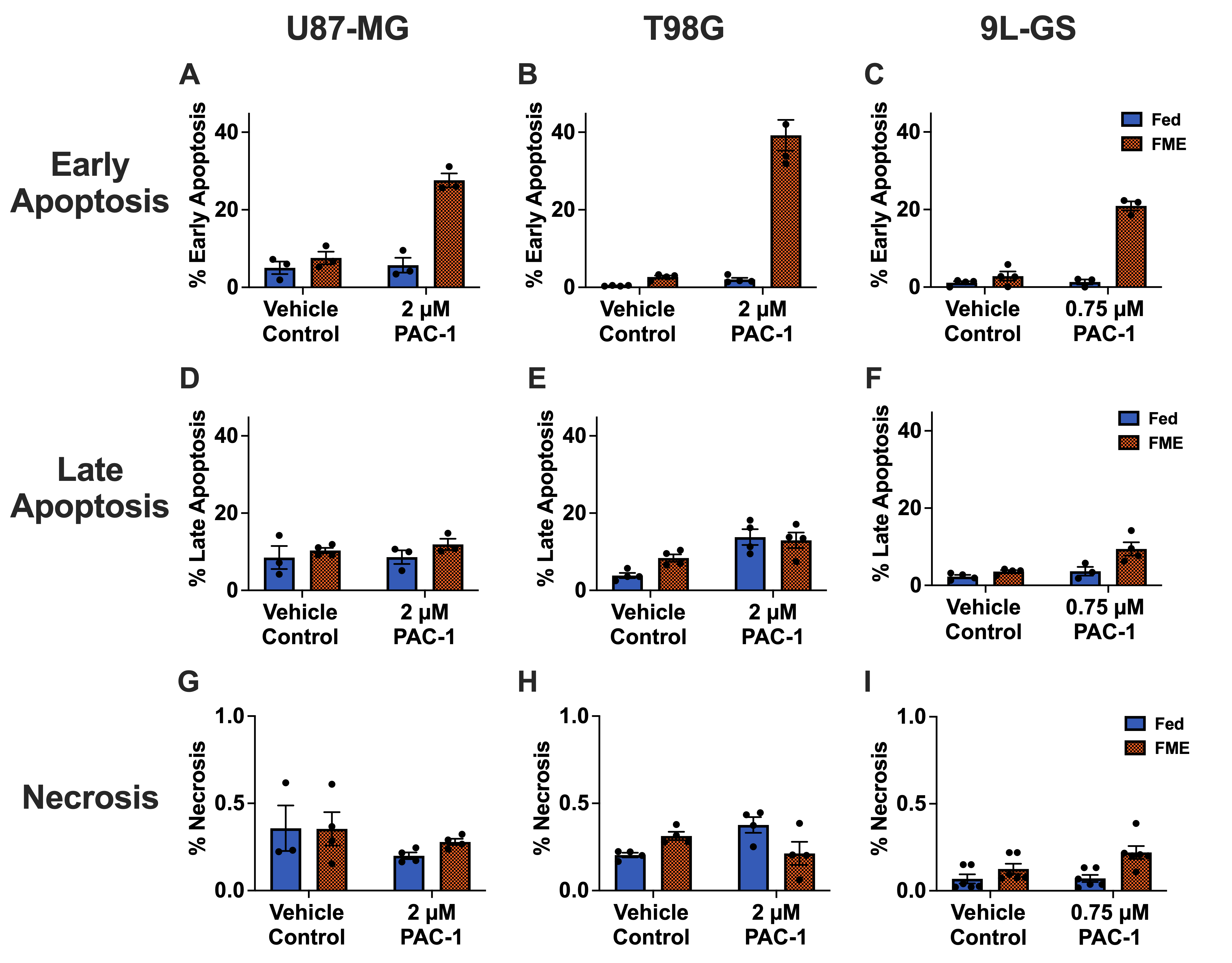
**

**Fig. S2. Percentage of early and late apoptosis and necrosis after PAC-1 treatment in Fed or FME conditions.** The early apoptotic population (**A-C**) was determined as being Annexin-V positive and propidium iodide (PI) negative, whereas the late apoptotic population (**D-F**) was defined as being Annexin-V and PI positive. The cells undergoing necrosis (**G-I**) were characterized as Annexin-V negative and PI positive. Population percentages were calculated relative to the total number of events per sample. The concentration of PAC-1 tested represents the FME IC_40_, obtained using Fig. 1. The results are presented as the mean $\pm$ SEM of at least three independent repeats containing a minimum of four technical replicates.

**Fig. S3. Representative U87-MG spheroid images after PAC-1 treatment in Fed or FME conditions and during recovery in Fed conditions.** The spheroids were initially grown in Fed conditions and treated in Fed or FME conditions, with the first image taken after 72 hours of PAC-1 treatment. Following treatment, the spheroids recovered in Fed conditions with the second image referring to 144 hours after the commencement of treatment or 72 hours into recovery. Bar represents 800 µm.

**Fig. S4. Representative 9L-GS spheroid images after PAC-1 treatment in Fed or FME conditions and during recovery in Fed conditions.** The spheroids were initially grown in Fed conditions and treated in Fed or FME conditions, with the first image taken after 72 hours of PAC-1 treatment. Following treatment, the spheroids recovered in Fed conditions with the second image referring to 144 hours after the commencement of treatment or 72 hours into recovery. Bar represents 800 µm.
